# Zinc Deficiency Associated With an Increase in Mortality in COVID-19 Patients: A Meta-Analysis

**DOI:** 10.7759/cureus.77011

**Published:** 2025-01-06

**Authors:** Chirag Raval, Spencer Z Rheingold, Antonio M Gordon, Patrick Hardigan

**Affiliations:** 1 Medical School, Nova Southeastern University Dr. Kiran C. Patel College of Allopathic Medicine, Davie, USA; 2 Department of Internal Medicine, University Health Care, Hialeah, USA; 3 Health Professions Division, Nova Southeastern University Dr. Kiran C. Patel College of Allopathic Medicine, Davie, USA

**Keywords:** covid, cytokine, deficiency, inflammation, zinc

## Abstract

The exact role of zinc in COVID-19-infected patients is not well understood. We examined the effects and outcomes of zinc deficiency on COVID-19-infected patients. We focused on patient outcomes: severity, symptomatology, and mortality.

The meta-analysis was performed to examine whether COVID-19-infected individuals suffered greater symptomology and mortality. Secondary outcomes explored included severity and hospital length of stay.

For mortality, we found that COVID-19-infected individuals with zinc deficiency had a greater risk of mortality than individuals without zinc deficiency (risk ratio (RR)=5.77; 95% confidence interval (CI): 3.48, 9.54; p=0.004). For symptomology, we found that COVID-19-infected individuals with zinc deficiency had a greater risk of symptomatology than individuals without a zinc deficiency (RR=1.39; 95% CI: 1.13, 1.70; p=0.020).

Zinc-deficient individuals are at a greater risk for mortality and symptomatology. Our findings further reinforce the importance of supplementation as a prophylactic agent against viral infections such as COVID-19.

## Introduction and background

The COVID-19 pandemic has caused and continues to challenge our healthcare system. Since March 2020, the cumulative death toll due to COVID-19 infections is approaching 1.2 million deaths [1]. To reduce the burden of disease on both patients and the healthcare system, effective preventative measures should be explored and discussed. Previous studies have analyzed patient populations and found that patients with lower levels of zinc have worse symptomatology, while other studies have found data that shows serum zinc does not influence various outcomes in COVID-19-infected patients. While it is widely understood that zinc plays a supportive role in the immune system, there is a lack of comprehensive data on the implications of zinc deficiency for COVID-19-infected individuals. Moreover, oversupplementation and disorganized supplementation carry the risk of suffering from side effects, adverse drug interactions, allergic reactions, and preventable long-term systemic damage. Therefore, it is imperative to understand whether zinc deficiencies can predispose COVID-19-infected patients to worse clinical outcomes. 

An estimated 17.3% of the world's population is at risk of inadequate zinc intake, and the World Health Organization (WHO) estimates that "zinc deficiency affects 31% with the prevalence rates ranging from 4% to 73% in various regions of the world's population. In developing countries, zinc deficiency is one of the ten significant factors contributing to the burden of disease" [2-4]. Zinc deficiency is associated with the impairment of numerous metabolic processes, reduced resistance to infections due to impaired immune functions, changes in skin and its appendages, and disorders of wound healing and hemostasis [5]. Additionally, a confirmed deficiency in elemental zinc is associated with the release of pro-inflammatory cytokines. These cytokines include IL-1β, IL-6, and TNF-α [6]. The overproduction of pro-inflammatory cytokines is a key part of the COVID-19 pathogenesis [7]. Standard care for COVID-19-infected patients include a combination of pharmaceuticals tailored for the patient's level of infection and a medley of supplements such as vitamins C and D and zinc [8]. Zinc supplementation has been a part of a number of therapies, as zinc has been found to have antiviral properties [9]. Furthermore, Barnard et al. as well as Pormohammad et al. showed that increased intracellular zinc concentrations decrease SARS-CoV-2 replication [10-12]. 

While the world continues to recover from the pandemic, iterative mutations of COVID-19 and other viral pathogens such as the zoonotic orthopoxvirus monkeypox remain significant threats to both society and the healthcare system. Hence, a meta-analysis was performed to give insight into the existing literature and understand how a patient's zinc levels can affect their hospital survivability and symptomatology. This study aims to establish and reinforce the distinct and critical role of zinc supplementation in preventing viral infection by analysis of existing literature and data. Additionally, our findings stress the importance of adequate zinc consumption, especially for at-risk patient populations.

## Review

Search strategy

**Figure 1:**
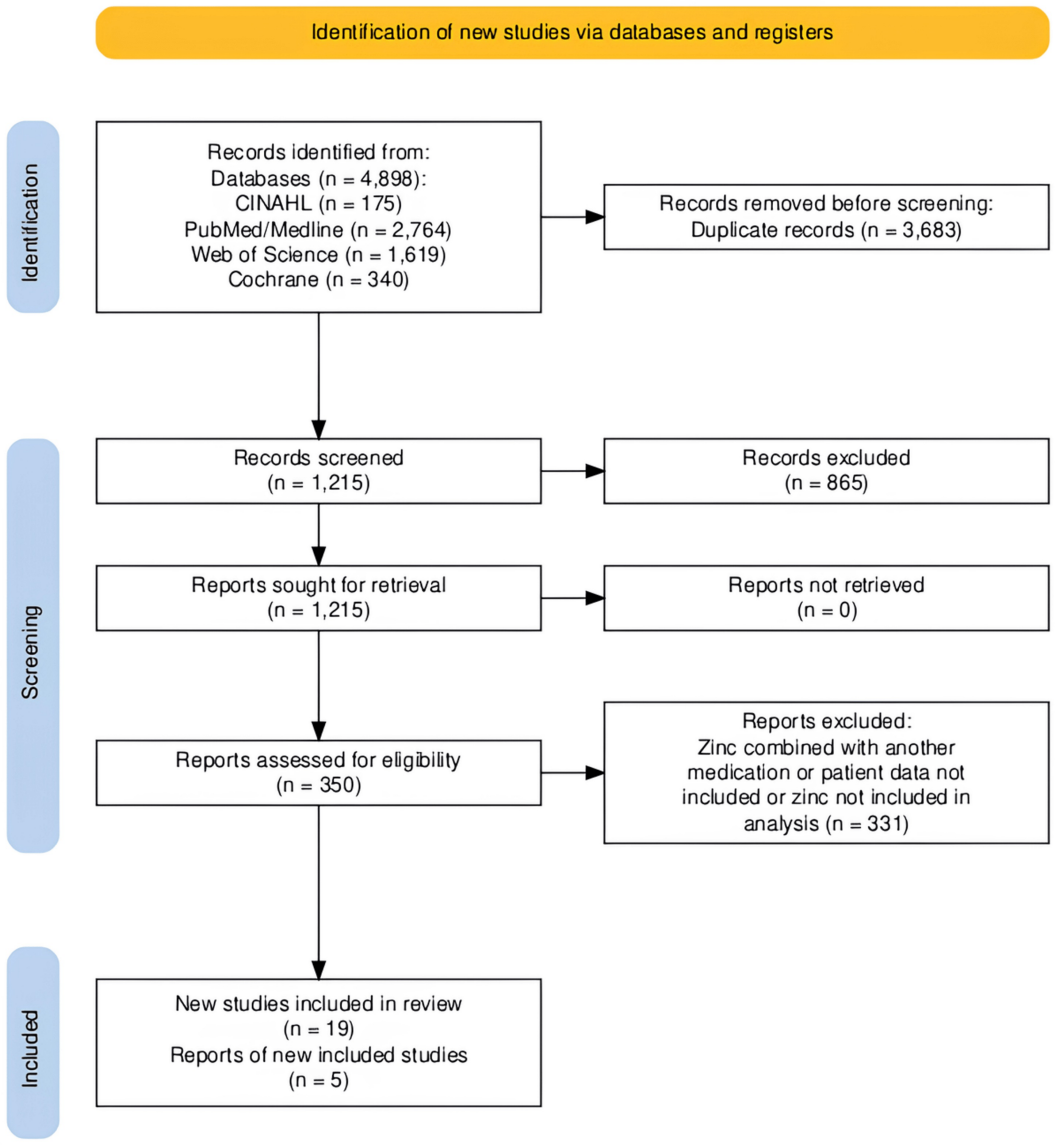
Identification of studies via databases (n=4). Records were retrieved from PubMed/MEDLINE, Cochrane, Web of Science, and CINAHL Complete.

This meta-analysis was carried out in accordance with the Preferred Reporting Items for Systematic Reviews and Meta-Analyses (PRISMA) guidelines [13]. Furthermore, this meta-analysis was constructed from a search by two authors, C.R. and S.R. A manual search was completed for this study. PubMed/MEDLINE, Cochrane, Web of Science, and CINAHL Complete were independently searched (Figure 1). For each database, records were retrieved using the following search terms: "zinc AND covid", "zinc AND sars-cov-2", "zinc AND COVID-19", and "zinc AND coronavirus". The references of the records that are included in our study were also reviewed. Our search was broadened to papers in English and other languages. Translated versions of the papers not in English were reviewed.

Inclusion and exclusion criteria

Using the PECO/PICO (population, exposure/intervention, comparison/control, and outcome) strategy, the studies that meet the following criteria were included in the study [14].

Inclusion Criteria

Populations: Subjects participated in studies that assessed the impact of zinc deficiency or low levels on COVID-19 infection.

Exposure/intervention: Exposure/intervention was between the zinc-deficient group and the control group (those with adequate zinc levels).

Study outcomes: Outcomes were mortality, hospital length of stay, and severity with regard to short-term symptomology and long-term symptomatology.

Exclusion Criteria

Excluded were studies with no accessible full text, studies that did not report specific outcomes quantitatively, and abstracts, comments, reviews, posters, and editorial reviews.

Data extraction

After performing an in-depth review of the records screened, raw data was extracted in a uniform fashion with a shared reporting system among two authors, C.R. and S.R. If either author disagreed or was unsure about a certain record, a third party, P.H., was available to act as a mediator. Our raw data included the following: author, year, serum zinc level, symptomology and severity, mortality, length of stay, and treatment outcomes. 

Risk of bias assessment

The risk of bias was assessed on a consensus three-point Likert scale (high, some concerns, and low) using the following criteria: bias due to randomization, bias due to deviations from intended interventions, bias due to missing data, bias due to outcome measurement, and bias due to selection of reported result. No papers were included or excluded based on these criteria.

Method

The meta-analysis was conducted through R Statistical Software, Version 4.2.1 (R Foundation for Statistical Computing, Vienna, Austria (https://www.R-project.org/)). Heterogeneity was evaluated by calculating the I^2^ index. I^2^ values less than 25%, 25-50%, 50-75%, and 75-100% were homogeneous or had low, medium, and high heterogeneity levels, respectively. The random-effects model was applied if the I^2^ value is >50%, while the fixed-effects model was applied if the I^2^ value is <50%. The combined risk ratio (RR) with the corresponding 95% confidence interval (CI) was used to assess the relationship between zinc deficiency and mortality and zinc deficiency and symptoms among COVID-19-infected individuals. Both mortality and symptomatology were operationalized as yes/no variables.

Results

From the initial literature search conducted, 4898 articles were selected based off of titles or abstracts alone. Duplicates were removed as well. After manual reference list searches and full-article reviews, four articles were selected to be included in the mortality analysis and two in the symptomatology analysis. We noticed in the mortality results a heterogeneity as measured by an I^2^ close to 0%, indicating that most variability in effect size estimates is due to sampling error within studies. For the symptomatology analysis, we had an I^2^ as very low as 0%. We employed the random-effects model on the assumption that any primary study result is influenced by unsystematic influences (therefore "random-effects" model). In case of the existence of true heterogeneity in our study, it may be that there are omitted systematic moderators of the effect of interest or as aforementioned unsystematic influences.

Zinc Levels and Mortality

**Figure 2:**
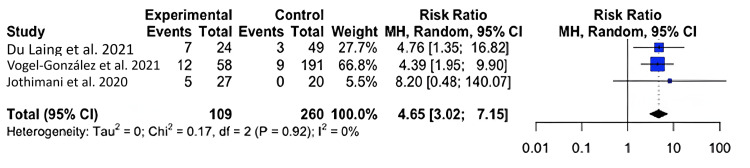
Forest plot for the risk difference of mortality for COVID-19-infected patients with and without a zinc deficiency.

For mortality, we found that COVID-19-infected individuals with zinc deficiency had a greater risk of mortality than individuals without zinc deficiency (RR=5.77; 95% CI: 3.48, 9.54; p=0.004) (Figure 2).

Zinc Levels and Symptomatology

**Figure 3:**
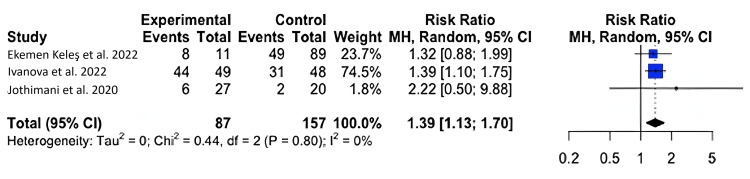
Forest plot for the risk difference of symptomatology for COVID-19-infected patients with and without a zinc deficiency.

For symptomology, we found that COVID-19-infected individuals with zinc deficiency had a greater risk of symptomatology than individuals without a zinc deficiency (RR=1.39; 95% CI: 1.13, 1.70); p=0.020) (Figure 3).

Zinc Levels and Severity

**Figure 4:**
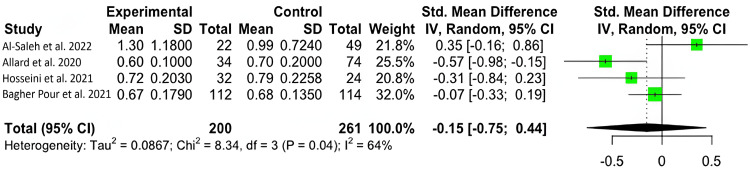
Forest plot for the standardized mean difference in zinc levels between patients experiencing severe and mild symptoms.

We found significant variability and used the random-effects model (I^2^=64%; 95% CI: 0%, 87.8%). No significant difference was found in zinc levels between those with severe (M=41.67 μmol/L; SD=33.00 μmol/L) and moderate (M=65.25 μmol/L; SD=38.38 μmol/L) COVID-19 symptoms, with a mean difference of -0.15 μmol/L (95% CI: -0.75 μmol/L, 0.44 μmol/L; p=0.4707) (Figure 4).

**Figure 5:**
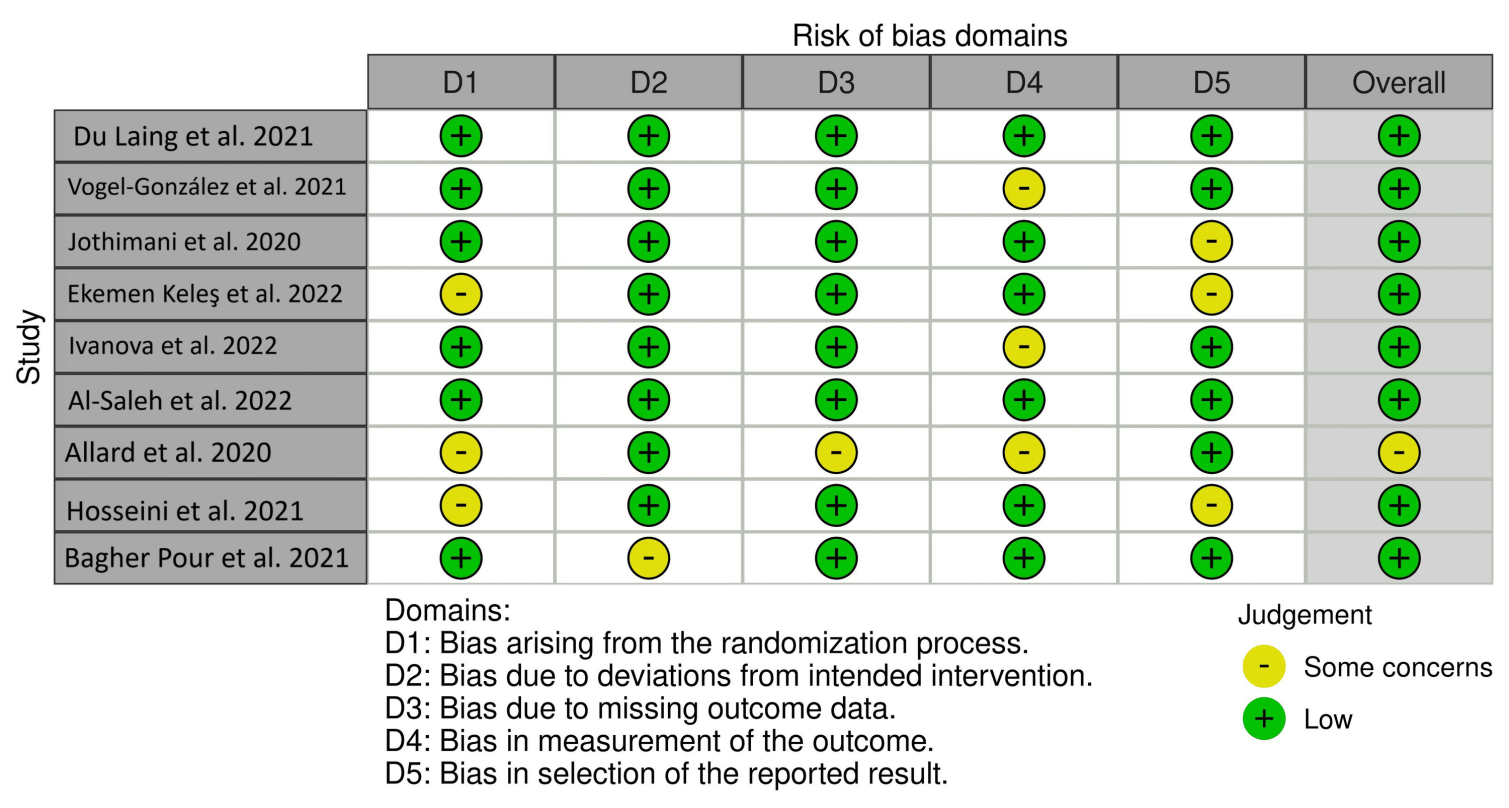
Risk of bias traffic light plot.

Figure 5 depicts the risk of bias traffic light plot.

Discussion

Our results show that zinc-deficient individuals are at a greater risk for mortality and symptomatology. The National Institutes of Health Office of Dietary Supplements recommended that the daily allowance of zinc for males and females above 19 years of age is 11 mg and 8 mg, respectively. A normal serum zinc level is defined as 80-120 µg/dL [15]. In each of our studies, patients had serum zinc concentrations at or below what is considered to be a normal serum zinc level. In Du Laing et al., three of five patients who died had the lowest levels of zinc in their study population as well as deficiencies in other trace minerals [16]. Non-survivors had dropped below the threshold for what was defined as a severe zinc deficiency (less than 660 µg/L) [15-16]. Vogel-González et al. found that a severe deficiency in zinc (less than 500 µg/L) was associated with a significantly higher level of mortality, as well as a longer time to recovery [17]. Jothimani et al. recruited 47 COVID-19-infected individuals and found that zinc-deficient individuals had a higher rate of complications and symptomatology and increased mortality [18]. We were not able to find a significant correlation between COVID-19 disease severity and zinc deficiency, though a higher level of symptomatology and the presence of symptoms were seen in zinc-deficient individuals [19-22]. More patient data is necessary to reach a definitive conclusion on the impact of zinc on infection severity. Yet, our meta-analysis found that, across multiple studies, patient outcomes were unfavorable among patients with zinc deficiency [23-24].

Zinc serves important roles in both the innate and adaptive immune systems [25-26]. In the innate nonspecific immune system, zinc modulates signaling molecules and regulates signaling pathways. It also regulates protein tyrosine kinases (PTKs) and protein kinase C (PKC) enzymes. Zinc is an important structural component of PKCs [27-28]. It is a major regulatory agent of the transcription factor nuclear factor kappa B (NF-κB). NF-κB initiates the production of pro-inflammatory cytokines such as TNF-α, IL-1β, IL-6, and IL-8 [27]. Zinc modulates NF-κB signaling through two zinc finger proteins: protein A20 and peroxisome proliferator-activated receptor alpha (PPAR-α) [27,29-30]. Both of these proteins are negative regulators induced by zinc and zinc supplementation. Induction of protein A20 leads to decreased levels of IL-1β and TNF-α through the decreased activity of NF-κB. Induction of PPAR-α stops NF-κB from binding DNA [27]. In addition to zinc's pivotal role in inflammation regulation, zinc is essential for the development and adequate functioning of each innate immune system cell type. For instance, granulocytes such as polymorphonuclear neutrophils (PMNs), the most abundant circulating blood leukocyte, are affected by zinc deficiencies [27,31]. Specifically, zinc deficiency causes impaired chemotaxis and phagocytosis and impairs the oxidative burst. Furthermore, zinc is required for granule mobilization.

Zinc plays a protective role in the innate immune system by acting as a cofactor for the superoxide dismutase family of enzymes, which are responsible for counteracting damage from oxidative stress [32]. It also plays a role in the differentiation and proliferation of macrophages. It plays both activating and inhibiting roles suggesting optimal monocyte and macrophage activity is mediated by homeostatic concentrations of zinc. As for mast cells, zinc is thought to play a role in the degranulation process as well as serve as part of the pathogen toxin [27]. This has been shown through prolonged signaling when zinc supplementation occurs [33]. Natural killer (NK) cells play an important role in fighting viral infection. When MHC-1 is downregulated, there is a lack of killer inhibitory receptor engagement. This in turn causes the infected cell to be processed by the NK cell. Under zinc-deficient conditions, the function and production of NK cells are diminished [27,34]. Additionally, NK cells are also affected by the downregulation of IL-2 during zinc-deficient conditions [35]. Barrier defense plays a large role in defense against pathogens. Zinc helps to support barrier defense against COVID-19, as the epithelial lining of the lungs serves as the primary defense point against the site of initial infection when inhaling the COVID-19 virus [36]. Furthermore, the lungs are the site of ACE2 expression, which is the primary method by which COVID-19 gains entry into the cell [37]. Zinc-deficient conditions cause the degradation of the tight and adherens junctions, weakening the defense against the virus and other pathogens.

Zinc supports the adaptive immune system. Not only does zinc support both B- and T-cell development, but zinc deficiencies result in reduced T-cell counts [27,38]. This is due to thymus atrophy and reduced thymulin activity in zinc-deficient conditions. Zinc stabilizes the signaling complex for T-cell activation [27]. It is important for Th1 differentiation through the upregulation of IFN-γ and enhances the capacity to produce Treg [39]. In summary, zinc ensures an adequate immune response to infection by playing an essential supportive role in the production and maintenance of both innate and adaptive immune responses.

Zinc has been shown to perform a plethora of supportive and regulatory actions in the immune system, and the data show that zinc deficiency shows increased symptomatology and mortality. Avoiding zinc deficiency is important for preventative and infection mitigation practices. The current literature and our data show that adequate levels of zinc can provide accessible and practical protection against infection and infectious processes when taken in a reasonable/bioavailable fashion. However, our study can only provide insight into the current data on zinc deficiencies and COVID-19. To definitively confirm these assertions, additional randomized, double-blind, placebo-controlled studies should be performed with newer variants of COVID-19 and other relevant viral threats to gain a greater understanding of the necessity of maintaining zinc homeostasis.

## Conclusions

Zinc-deficient patients suffering from COVID-19 may be at a greater risk for mortality and symptomatology. Our findings further reinforce the importance of supplementation as a prophylactic agent against viral infections such as COVID-19. Though no association was found between zinc and the severity of COVID-19 symptomatology, the present study confirms the importance of zinc as a possible protective agent against COVID-19 symptomatology, regardless of severity and, subsequently, mortality.
